# Alpha-Synuclein Oligomers—Neurotoxic Molecules in Parkinson's Disease and Other Lewy Body Disorders

**DOI:** 10.3389/fnins.2016.00408

**Published:** 2016-09-05

**Authors:** Martin Ingelsson

**Affiliations:** Rudbeck Laboratory, Department of Public Health/Geriatrics, Uppsala UniversityUppsala, Sweden

**Keywords:** alpha-synuclein oligomers, Lewy body disease, toxicity, biomarker, propagation, therapeutics

## Abstract

Adverse intra- and extracellular effects of toxic α-synuclein are believed to be central to the pathogenesis in Parkinson's disease and other disorders with Lewy body pathology in the nervous system. One of the physiological roles of α-synuclein relates to the regulation of neurotransmitter release at the presynapse, although it is still unclear whether this mechanism depends on the action of monomers or smaller oligomers. As for the pathogenicity, accumulating evidence suggest that prefibrillar species, rather than the deposits *per se*, are responsible for the toxicity in affected cells. In particular, larger oligomers or protofibrils of α-synuclein have been shown to impair protein degradation as well as the function of several organelles, such as the mitochondria and the endoplasmic reticulum. Accumulating evidence further suggest that oligomers/protofibrils may have a toxic effect on the synapse, which may lead to disrupted electrophysiological properties. In addition, recent data indicate that oligomeric α-synuclein species can spread between cells, either as free-floating proteins or via extracellular vesicles, and thereby act as seeds to propagate disease between interconnected brain regions. Taken together, several lines of evidence suggest that α-synuclein have neurotoxic properties and therefore should be an appropriate molecular target for therapeutic intervention in Parkinson's disease and other disorders with Lewy pathology. In this context, immunotherapy with monoclonal antibodies against α-synuclein oligomers/protofibrils should be a particularly attractive treatment option.

## Lewy pathology/α-synuclein

**Figure 1:**
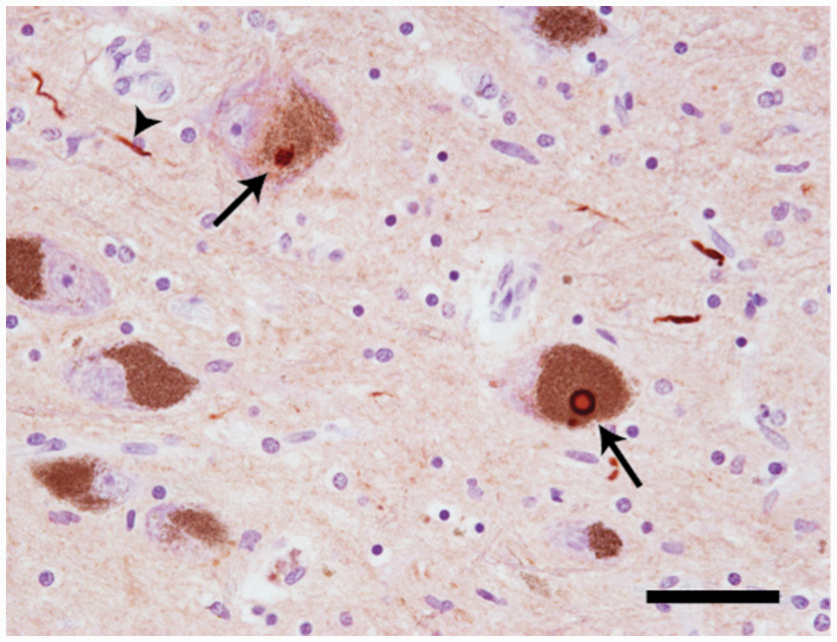
**Neuropathology of Lewy body disorders**. Lewy bodies and Lewy neurites (arrows) in substantia nigra from a PD patient, stained with a polyclonal antibody directed against α-synuclein amino acids 126–135 (20 × magnification). The arrows point toward Lewy bodies. The arrowhead points toward a Lewy neurite. Scale bar = 50 μm. Photo: Leire Almandoz Gil, Uppsala University.

At autopsy, widespread neuronal loss can be seen in the brain stem and neocortex of patients with Parkinson's disease (PD), dementia with Lewy bodies (DLB), multiple system atrophy (MSA) and the Lewy body variant of Alzheimer's disease (LBAD). Collectively, these diseases are referred to as Lewy body disorders. Intracellular protein inclusions, known as Lewy bodies and Lewy neurites, can be seen in a portion of the surviving cells (Figure 1). For PD, DLB, and LBAD, most of such deposits can be found in neurons, whereas in MSA they are mainly displayed in glial cells (reviewed in Braak and Braak, 2000).

The principal component of the intraneuronal and intraglial inclusions is α-synuclein, a protein of 140 amino acids that is ubiquitously expressed in neurons (Jakes et al., 1994). Whereas, α-synuclein is intrinsically disordered in the cytosol, it adopts an alpha-helical conformation when it becomes bound to cellular membranes (Weinreb et al., 1996; Kim, 1997; Chandra et al., 2003; Fauvet et al., 2012; Burre et al., 2013). The normal function of α-synuclein has only been partly understood, but because of its principal localization to the synaptic region it has been suggested to play a role in the regulation of neurotransmitter release (Burre et al., 2010). Mainly, it has been demonstrated that α-synuclein can interact with the components of the SNAP (Soluble NSF Attachment Protein) Receptor (SNARE) complex in the presynapse, e.g., synaptobrevin-2 (Burre et al., 2010), synapsin III (Zaltieri et al., 2015) and rab3A (Chen et al., 2013; reviewed in Burre, 2015). In addition, α-synuclein can be found also at other cellular locations, such as the cytosol and mitochondria (Chinta et al., 2010).

More recently, it has been demonstrated that α-synuclein also can be secreted and transferred to nearby cells (Li et al., 2008; Hansen et al., 2011). Such cell-to-cell transfer provides an attractive explanation for the hierarchical spreading of Lewy pathology within the central nervous system (CNS) (Braak et al., 2003) and to the more recent question why the presence of α-synuclein in neuronal plexa of the intestinal wall seems to precede the formation of brain inclusions (Braak et al., 2006). The underlying mechanisms for such propagation of pathological α-synuclein have only been partially elucidated, but seem to involve retrograde transport (Holmqvist et al., 2014) as well as release via exosomes and other extracellular vesicles (Emmanouilidou et al., 2010; Bellingham et al., 2012; Danzer et al., 2012).

## Features of physiological α-synuclein

Different studies have come to different conclusions regarding the properties of physiological α-synuclein. One research group has proposed that the normal cellular state of the protein is a tetrameric complex, which needs to dissociate before the monomers can start to aggregate (Bartels et al., 2011; Dettmer et al., 2013). However, in a large multicenter study it could be demonstrated that α-synuclein predominantly exists as an unfolded monomer, at least in the CNS (Fauvet et al., 2012). Moreover, a recent investigation lends further support to this notion and also provides detailed information of the structure and post-translational modifications of the α-synuclein monomer (Theillet et al., 2016). Most importantly, this study indicates that physiological α-synuclein monomers are amino-terminally acetylated and thereby adopt a compact conformation. As a consequence, mid region epitopes critical for the assembly of α-synuclein into multimers should then be protected from the cytoplasmic exposure needed for spontaneous aggregation (Theillet et al., 2016).

## The α-synuclein cascade hypothesis

In addition to the clinicopathological correlation, i.e., the finding of α-synuclein-containing pathology in affected brain areas, genetic evidence strongly support the α-synuclein cascade hypothesis—that the formation of aggregating α-synuclein species precedes synaptic dysfunction and subsequent neuronal death (reviewed in Houlden and Singleton, 2012). As for several other neurodegenerative diseases, molecular genetic findings have been crucial to further our understanding of the pathophysiology. To date, six point mutations (Polymeropoulos et al., 1997; Conway et al., 1998; Kruger et al., 1998; Zarranz et al., 2004; Appel-Cresswell et al., 2013; Lesage et al., 2013; Proukakis et al., 2013), as well as duplications (Chartier-Harlin et al., 2004) and triplications (Singleton et al., 2003) of the α-synuclein gene have been identified. All of these mutations lead to early-onset forms of familial α-synucleinopathy, with a seemingly very high penetrance (reviewed in Houlden and Singleton, 2012).

At least two of the α-synuclein mutations, A30P and A53T, were early shown to promote an increased formation of large soluble oligomers—or protofibrils—which was suggested to be their pathophysiological mechanism (Conway et al., 2000). These observations have been followed by numerous *ex vivo* and *in vivo* studies, supporting that the oligomers exert more pronounced neurotoxic effects than the fibrils *per se*. Interestingly, a recent study identified different structures between mutant and wild-type α-synuclein oligomers, suggesting that pathology may be related not only to the amount of oligomers but also to the structural and functional properties of the species formed (Tosatto et al., 2015).

**Figure 2:**
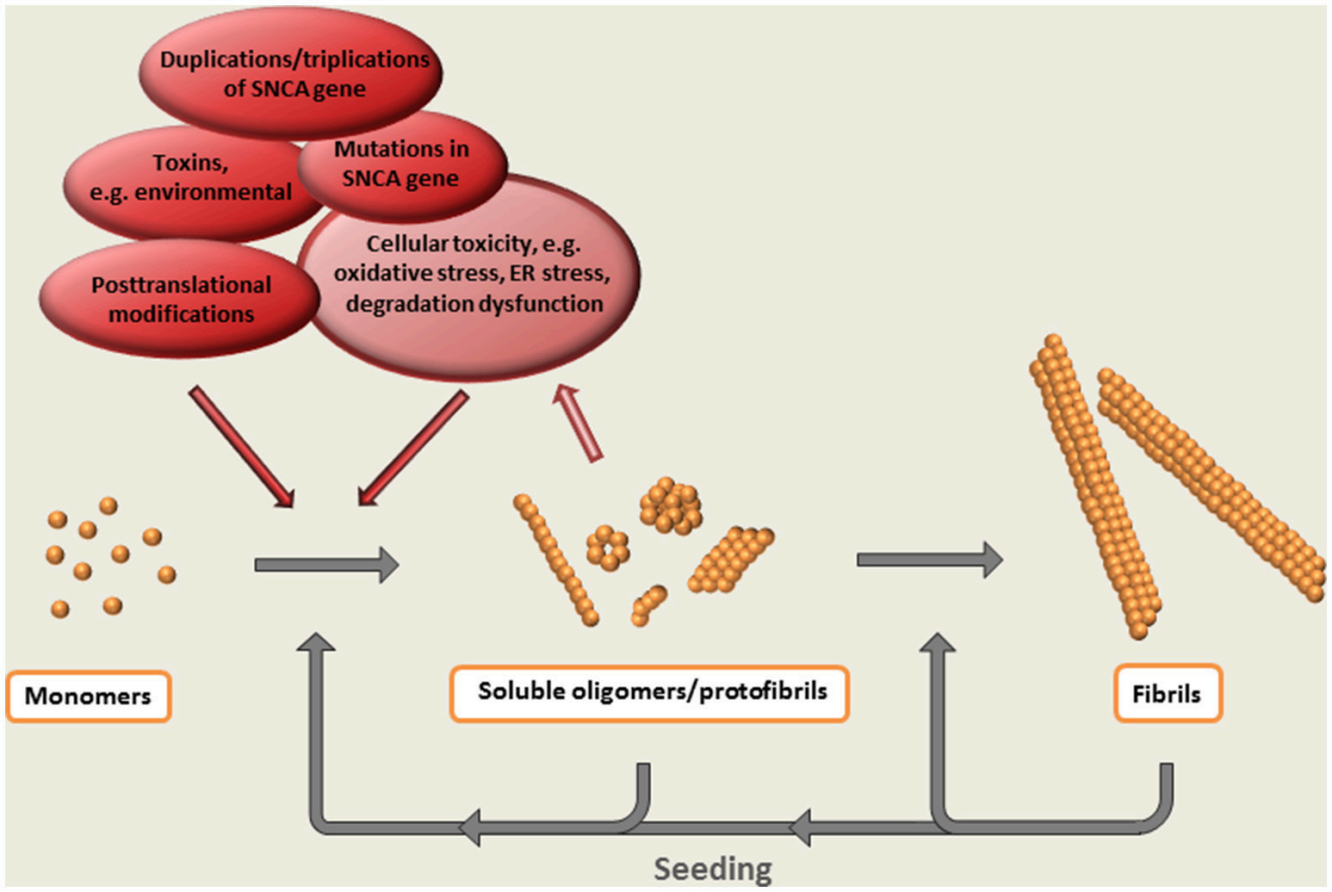
**The α-synuclein cascade hypothesis**. The aggregation of α-synuclein presumably starts with a conformational shift of the monomeric protein, followed by the step-wise formation of larger multimeric protein species. Evidence suggest that soluble oligomeric / protofibrillar aggregates are the most neurotoxic forms of α-synuclein. Such species, as well as the ready-formed fibrils, may also potentiate pathology by acting as seeds for the formation of additional aggregates.

Thus, the formation of oligomeric species can be regarded as a central event in the α-synuclein cascade hypothesis (Figure 2). Whereas, the appearance of these intermediately sized, soluble aggregates precede the formation of Lewy bodies and Lewy neurites (*on pathway* oligomers)—or if they are arrested in an oligomeric state (*off pathway* oligomers) has not been clarified.

## Alpha-synuclein oligomers

Increased levels of differently sized α-synuclein oligomers have been measured in brains with Lewy pathology compared to brains from non-diseased individuals (Sharon et al., 2003; Kramer and Schulz-Schaeffer, 2007; Paleologou et al., 2009). Furthermore, several studies have described elevated levels of oligomeric α-synuclein in cerebrospinal fluid (CSF) in PD patients compared to control subjects (Tokuda et al., 2006; Park et al., 2011; Aasly et al., 2014; Hansson et al., 2014; Parnetti et al., 2014). All of the CSF studies have adopted an ELISA based on the monoclonal α-synuclein antibody 211 as capture antibody and its biotinylated version as reporter antibody. Such an approach avoids detection of monomeric α-synuclein, but cannot distinguish between the different multimeric forms. Additional proof of concept studies have demonstrated the feasibility of using antibodies that can selectively measure particular disease-related oligomeric species (Sierks et al., 2011; Brannstrom et al., 2014; Unterberger et al., 2014), but no larger case-control studies based on such antibodies have so far been reported.

In addition to these direct clinical and clinico-pathological observations, a large number of studies have investigated the physiological effects of α-synuclein oligomers on various cellular, tissue and animal models. For *ex vivo* studies large α-synuclein oligomers (with a size ≥ 600 kDa) can be generated from recombinant protein. Although, the concentration of protein needs to be high (≥ 200 μM), the oligomeric yield is typically low (less than 15% of the starting material, Lashuel et al., 2002). Also, the resulting oligomers are typically prone to degradation. However, by using certain molecular agents, more structurally stable α-synuclein oligomer can be induced (Lashuel et al., 2002).

## *In vitro* generation of α-synuclein oligomers

Several protocols, by which recombinant α-synuclein can be oligomerized, have been described. For example, polyphenol(-)-epigallocatechin gallate, baicalein, nicotine, dopamine, H_2_O_2_ and 3,4-dihydroxyphenylacetic acid have all been used to promote α-synuclein oligomerization *in vitro* (Cappai et al., 2005; Ehrnhoefer et al., 2008; Hong et al., 2008, 2009; Zhou et al., 2009, 2010; Bieschke et al., 2010). In addition, oxidative modification by e.g., methionine oxidation of α-synuclein also induces oligomer formation (Uversky et al., 2002). However, most of these modified oligomers lack a distinct secondary structure and have an *off pathway* character. Typically, they either inhibit the formation of fibrils or disassemble already formed fibrils and are usually non-toxic.

Various molecules involved in oxidative stress have been described to induce α-synuclein oligomerization. Mainly, the reactive aldehyde 4-hydroxy-2-nonenal (HNE) was found to covalently modify α-synuclein *in vitro* and thereby induce stable β-sheet-rich oligomers with neurotoxic properties (Qin et al., 2007). Also other aldehydes, such as acrolein and 4-oxo-2-nonenal (ONE), have been shown to induce α-synuclein oligomerization (Shamoto-Nagai et al., 2007; Näsström et al., 2009). Although, the direct link between oxidative stress and α-synuclein aggregation is unknown, short-lived reactive oxygen species, known to initiate lipid peroxidation of polyunsaturated fatty acids, have been shown to be present in neuronal cell membranes. Such peroxidation can lead to the formation of reactive aldehydes which, in addition to being cytotoxic themselves, can bind covalently both to α-synuclein and to other proteins and thereby alter their normal structure and function (Esterbauer et al., 1991).

## Proposed mechanisms behind α-synuclein mediated toxicity

**Figure 3:**
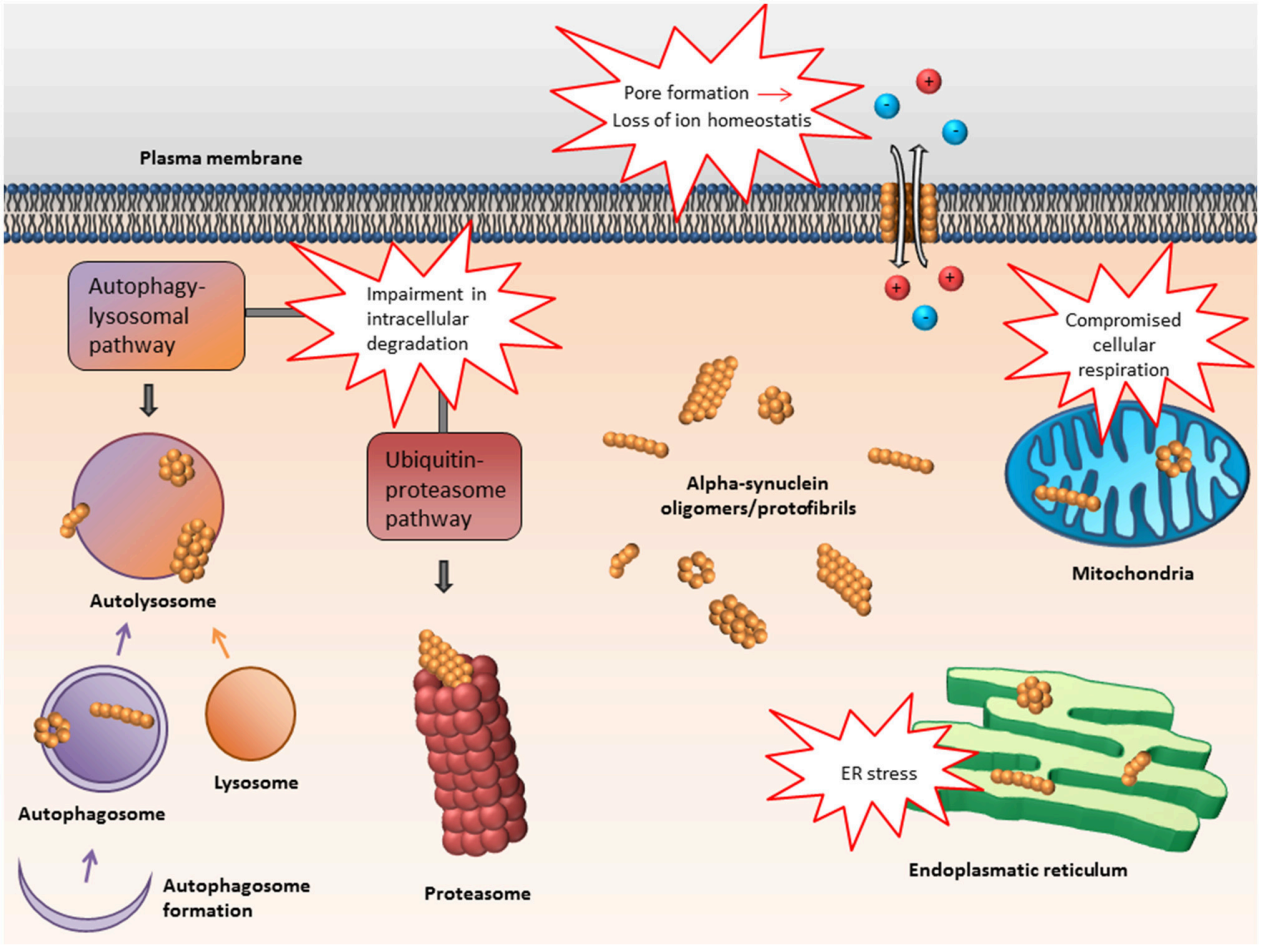
**Proposed intracellular targets for α-synuclein mediated toxicity**. Alpha-synuclein oligomers may mediate toxicity via several intracellular targets. Mainly, impairment of various protein degradation pathways as well as damage of the mitochondria and endoplasmic reticulum have been suggested.

**Figure 4:**
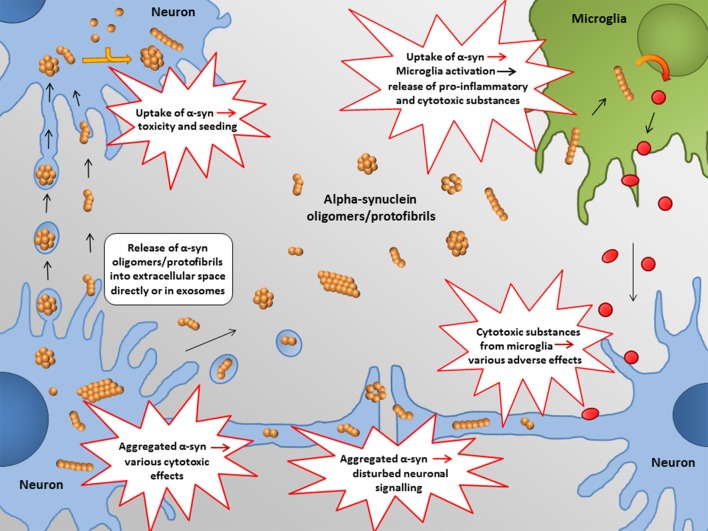
**Proposed extracellular targets for α-synuclein mediated toxicity**. Recent studies indicate that α-synuclein oligomers/protofibrils can propagate between neurons as either free floating proteins or via exosomes / other extracellular vesicles. Release and uptake of such species seem to occur either at the cell body or at the synapse. Also glial cells, such as astrocytes, may be involved in this process.

In the following, some of the proposed intra- and extracellular consequences of toxic α-synuclein oligomers will be discussed. Their potential targets and effects are summarized in Figures 3, 4.

### General cellular toxicity

Using various forms of *in vitro* generated α-synuclein, several *ex vivo* and *in vivo* studies have found that such species have pronounced toxic effects on cells. In one study it was demonstrated that *in vitro* generated large α-synuclein oligomers could induce cellular pathology (Danzer et al., 2007). Upon generation and characterization of various types of oligomers, all were shown to have various damaging effects on cells in culture. One variant was proposed to induce cell death via disruption of cellular ion homeostasis by a pore-forming mechanism, whereas another was able to directly enter cells and cause increased protein aggregation (Danzer et al., 2007). A subsequent study used a cell model for α-synuclein oligomerization and found that induction of oligomers resulted in increased cell toxicity (Outeiro et al., 2008).

When exposing lower organisms, like *c. elegans* and *d. melanogaster*, to *in vitro* generated α-synuclein oligomers, pronounced neurotoxic effects could be demonstrated (Karpinar et al., 2009) Also, in a study on transgenic mice it was found that mice expressing the artificial α-synuclein variants *E57K* and *E35K*, engineered to promote oligomer formation, displayed a more severe loss of dopaminergic neurons as compared to regular α-synuclein transgenic mice, overexpressing wild-type α-synuclein (Winner et al., 2011).

### Compromised cell membrane integrity

Several observations suggest that α-synuclein oligomers, similar to certain protein toxins, can disrupt cellular homeostasis by creating pores in the cell membrane. A disruption of the outer lipid bilayer can cause an increased permeability and influx of ions from the extracellular space (Volles et al., 2001; Volles and Lansbury, 2002; Danzer et al., 2007). In one of these studies, oligomers with such properties could be induced by incubating recombinant α-synuclein for 6 days in a sodium phosphate buffer, followed by exposure to iron. The formed oligomers were shown to form pores in a synthetic bilayer assay. Moreover, when exposing these protofibril-shaped species to primary cortical neurons they caused a depolarization of the cell membrane, presumably due to iron fluxes through the membrane.

### Synaptic toxicity/deficient neuronal signaling

Since evidence suggest that α-synuclein plays a role in the regulation of the SNARE-complex at the synapse (reviewed in Burre, 2015), it has been speculated that oligomeric α-synuclein may perturb this function and thereby cause a direct synaptotoxic effect (Choi et al., 2013).

In one study on hippocampal brain slices from rats, exogenously added α-synuclein oligomers were found to have a negative impact on neuronal signaling (Diogenes et al., 2012). Tissues that had been pre-incubated with α-synuclein oligomers displayed an increase in synaptic transmission leading to a suppression of long term potentiation, as determined by extracellular recordings of excitatory postsynaptic potential (EPSPs) from Schaeffer-collaterals/CA1 glutamatergic fibers. However, when monomeric or fibrillar α-synuclein species were added at similar concentrations, no such effects could be observed. The synaptic dysregulation seemed to depend on the presence of N-Metyl-D-Aspartate (NMDA) receptors, as the effects could be avoided by selectively blocking this glutamate receptor subtype (Diogenes et al., 2012).

In a more recent study, two different types of oligomers were generated either by polymerization of monomers or by sonication of fibrils (Kaufmann et al., 2016). Although, the structure of these species differed somewhat, they were both found to have a negative impact on neuronal excitability as indicated by patch-clamp recordings of pyramidal neurons in neocortical brain slices from mouse (Kaufmann et al., 2016).

Alpha-synuclein oligomer-induced synaptic dysfunction has also been suggested *in vivo*. Extensive synaptic and dendritic loss, in parallel with a reduction in synapsin 1 and synaptic vesicles, could be demonstrated on mice expressing the artificial and oligomerization prone α-synuclein mutation E57K under the Thy-1 promoter (Rockenstein et al., 2014). Thus, these findings suggest that accumulating oligomeric α-synuclein may mediate early synapse-related pathology by disrupting synaptic vesicles in CNS neurons.

### Impairment in intracellular degradation

Failure in protein degradation pathways, such as the ubiquitin-proteasome system or the autophagy-lysosomal pathway, has in several investigations been implicated as a consequence of α-synuclein oligomer formation. One study found increased cellular toxicity, as measured by the release of adenylate kinase and increase of the apoptotic marker caspase-3, upon lysosomal inhibition by bafilomycin, whereas activation of the lysosomes by rapamycin resulted in decreased toxicity (Klucken et al., 2012). Interestingly, the bafilomycin-induced toxicity was paralleled by a decrease in fibril formation, whereas the levels of insoluble smaller species increased, suggesting that α-synuclein oligomers may have mediated the toxic effects observed (Klucken et al., 2012).

Defects in phagocytosis have also been suggested as a disease-causing mechanism for α-synucleinopathies. In particular, uptake and subsequent degradation of α-synuclein oligomers by microglia and astrocytes may be an important mechanism for the prevention and/or clearance of pathology. Such an impairment may be related to various inherent factors and lead to disease susceptibility in certain individuals. Recently, it could be demonstrated that cultured microglia from adult mice displayed a less efficient uptake and clearance of oligomeric α-synuclein as compared to younger mice. Similar effects could be demonstrated when analyzing peripheral monocytes from older vs. younger human subjects (Bliederhaeuser et al., 2016). Taken together, these findings suggest that there might be an age-dependent deficit in the uptake and clearance of toxic α-synuclein by phagocytic cells.

### Mitochondrial toxicity

One study found that overexpression of the oligomer-promoting α-synuclein mutants *A53T* and *A30P* in human neuroblastoma cells augmented aggregation of α-synuclein (Parihar et al., 2009). Immuno-gold electron transmission microscopy showed localization of the α-synuclein aggregates within the mitochondria of overexpressing cells, which displayed decreased mitochondrial transmembrane potential as well as compromised cellular respiration (Parihar et al., 2009). More recently, it could be demonstrated that α-synuclein oligomers can prevent the import of proteins into the mitochondria of cultured cells by interacting with the translocase of the outer membrane 20 (TOM 20) and thereby disrupt its normal association with TOM 22. The functional relevance of such a mechanism was indicated by the finding of decreased amounts of Ndufs3, a complex I subunit of the respiratory chain, in the mitochondria of cells that had been exposed to oligomers, whereas no such effect could be seen for cells that had been subjected to monomers or fibrils of α-synuclein. Interestingly, it was also found that substantia nigra from PD brains displayed a pronounced molecular interaction between α-synuclein and TOM 20 as well as decreased levels of Ndufs3, as compared to control brains (Di Maio et al., 2016).

### Dysfunction of the endoplasmic reticulum

It has been shown that accumulation of α-synuclein oligomers/protofibrils in the endoplasmic reticulum (ER) leads to ER stress, which may contribute to neurodegeneration (Colla et al., 2012b). The same authors found that induction of the ER chaperones grp94, grp78 and PDI in affected neurons of the brain stem and spinal cord coincided with the onset of symptoms in an α*-synuclein A53T* transgenic mouse model (Colla et al., 2012a). Moreover, the study described data suggesting that salubrinal, an inhibitor of the eIF2α phosphatase, could attenuate the accumulation of toxic α-synuclein oligomers (Colla et al., 2012b) and reduce oligomeric microsomal accumulation in microsomes, vesicle-like structures formed from the ER, in treated mice (Colla et al., 2012a).

### Inflammatory responses

The role of inflammation in the neurodegenerative brain is only partly understood, but seems to involve both protective and damaging effects. Whereas, the removal of dead cells and debris depend upon phagocytosis by microglia and/or astrocytes an increased release of cytokines can, under certain circumstances, cause neuronal dysfunction and cell death. As for α-synuclein, aggregated extracellular species have been shown to activate microglia and thereby cause inflammation and degeneration of affected neurons (Zhang et al., 2005; Wilms et al., 2009). In a primary mesencephalic neuron-glia culture system, activation of microglia was found to enhance dopaminergic neurodegeneration induced by such α-synuclein species. Moreover, it was proposed that the glia-mediated enhancement of the neurotoxicity was depending on phagocytosis of α-synuclein, leading to the activation of NADPH oxidase and generation of reactive oxygen species (Zhang et al., 2005). In another study, subjecting cultured microglial cells to α-synuclein protofibrils, proinflammatory signaling pathways involving p38, ERK1/2 MAP kinases and NF-κB became activated. In addition, injection of α-synuclein protofibrils into substantia nigra of adult rats resulted in a profound activation of microglia together with adjacent neuronal cell loss, which could be attenuated by the MAP kinase inhibitor semapimod (Wilms et al., 2009).

Thus, several studies suggest that oligomeric/protofibrillar α-synuclein may exert some of its toxic effects by promoting reactions that could enhance the inflammatory response in the affected tissue. Especially microglia seem to be particularly prone to react upon exposure of such α-synuclein species and future studies will elucidate whether also astrocytes may contribute to damaging inflammatory responses elicited by α-synuclein oligomers/protofibrils.

### Seeding/cell-to-cell propagation

The growing realization that α-synuclein pathology can be propagated between cells imply that abnormal species can induce conformational changes and aggregation of physiological monomeric protein. Such seeding effects have been linked to α-synuclein oligomers. The first observation suggesting cell-to-cell spreading of α-synuclein was made at autopsy of PD brains that 10–15 years earlier had received fetal dopaminergic grafts as an experimental therapeutic approach. The researchers found that not only the host tissue, but also the graft, displayed Lewy pathology, suggesting that abnormal α-synuclein had been transferred from the affected host to the engrafted tissue (Kordower et al., 2008; Li et al., 2008; Mendez et al., 2008). Subsequent studies have investigated the underlying mechanisms and found that the α-synuclein oligomers seem to play a central role in these processes (Hansen et al., 2011).

*In vitro*-generated α-synuclein oligomers have been shown to induce transmembrane seeding of α-synuclein aggregation in primary neuronal cultures as well as in neuronal cell lines (Danzer et al., 2009). Thus, such extracellular α-synuclein oligomers seem to have the capacity to induce intracellular α-synuclein aggregation and could thus be responsible for spreading the disease between interconnected cells. The same research group could also demonstrate that α-synuclein oligomers are especially prone to get secreted in exosomes and that the presence of oligomers in such extracellular vesicles can mediate the intercellular spreading of α-synuclein pathology (Danzer et al., 2012).

Moreover, also non-neuronal cells may be involved in the cell-to-cell spreading of α-synuclein pathology. For example, cell culture-based studies suggest that astrocytes may internalize α-synuclein from neurons by endocytosis (Lee et al., 2010). Apart from generating inclusion bodies and induction of pro-inflammatory cytokines, such an uptake could promote further propagation of pathological α-synuclein.

## Toxic α-synuclein oligomers as a therapeutic target

Immunotherapy has emerged as a promising method to target α-synuclein pathology. When α-synuclein fibrils were peripherally administered on mice or rats expressing human α-synuclein, a reduced protein deposition in their brains could be seen (Masliah et al., 2005; Sanchez-Guajardo et al., 2013). Moreover, passive immunization with monoclonal α-synuclein antibodies on mice have also proven efficacious to both decrease pathology, to ameliorate symptoms and to prevent cell-to-cell propagation of pathology (Masliah et al., 2011; Bae et al., 2012; Games et al., 2014).

As accumulating evidence suggest that α-synuclein oligomers/protofibrils are responsible for the neurotoxicity in Lewy body disorders, researchers have begun to selectively target such species for immunotherapy, either by active or passive immunotherapy. By immunizing transgenic α-synuclein mice with certain peptides that mimic the c-terminus of α-synuclein and result in antibodies with oligomer-selective properties a reduced pathology as well as an improved outcome on motor tests were observed (Mandler et al., 2014).

We generated a large oligomeric form of α-synuclein and could demonstrate that this species had various toxic effects on *ex vivo* models (Nasstrom et al., 2011; Diogenes et al., 2012; Fagerqvist et al., 2013b). This α-synuclein species was also used as antigen to generate monoclonal antibodies with a pronounced oligomer/protofibril selectivity (Fagerqvist et al., 2013a). Initially, we used these antibodies to assess levels of α-synuclein oligomers/protofibrils in the CNS of α-synuclein transgenic mice and found that mice expressing high levels developed motor symptoms at an earlier stage than mice expressing low levels (Lindstrom et al., 2014). Next, we treated the mice with repeated intraperitoneal injections of one of these antibodies, resulting in reduced levels of α-synuclein in the CNS (Lindstrom et al., 2014). Ongoing studies will show whether such a treatment also can alleviate the motor and behavioral symptoms that the transgenic mice normally develop.

The prospect of using peripheral immunotherapy to target α-synuclein oligomers/protofibrils is a daunting task, regardless of whether one is taking the active or the passive immunization approach. Firstly, we still do not know by which means and via which mechanisms that the antibodies act. Secondly, the antibodies have to pass the blood brain barrier in sufficient amounts to exert an effect in the CNS. Thirdly, the antibodies probably have to reach into the cells (where most of the α-synuclein pathology is present) and at the same time avoid the normal, physiological forms of the protein. One attempt to increase the intracellular presence of therapeutic antibodies inside CNS is represented by virus-mediated delivery. In one recent study, lentivirus expressing oligomer-selective single chain α-synuclein antibodies were shown to be efficacious on transgenic mice, both with respect to pathology and motor symptoms (Spencer et al., 2016). Future studies with similar approaches will be needed to optimize delivery and expression of such “intrabodies.”

In spite of the remaining challenges and questions, sufficient evidence demonstrating the feasibility of α-synuclein immunization have been provided. The first clinical studies on humans, adopting α-synuclein immunotherapy, are already underway and the outcome of these will provide us with new knowledge that hopefully will help us in the development of a therapy that can efficiently alleviate both the pathology and the symptoms that patients with Lewy body disorders suffer from.

## Conclusions

Molecular genetic and biochemical evidence support the hypothesis that α-synuclein oligomers play a central role in the pathogenesis of Parkinson's disease and related disorders. Several intra- and extracellular mechanisms, by which such prefibrillar species of α-synuclein exert damaging effects, have been identified (Figures 3, 4). Apart from general cellular toxicity, mitochondrial stress, synaptic dysfunction and compromised cell membrane integrity are some examples of the proposed pathogenic mechanisms. Moreover, oligomers of α-synuclein can act as seeds for the formation of aggregates and also seem to be prone to transfer between cells. Taken together, the properties of α-synuclein oligomers indicate that they are particularly responsible for the propagation of pathology and that such species should be suitable targets for early therapeutic intervention in Parkinson's disease and related disorders.

## Author contributions

The author confirms being the sole contributor of this work and approved it for publication.

### Conflict of interest statement

The author declares that the research was conducted in the absence of any commercial or financial relationships that could be construed as a potential conflict of interest.
